# An Unusual Basal Therizinosaur Dinosaur with an Ornithischian Dental Arrangement from Northeastern China

**DOI:** 10.1371/journal.pone.0063423

**Published:** 2013-05-29

**Authors:** Hanyong Pu, Yoshitsugu Kobayashi, Junchang Lü, Li Xu, Yanhua Wu, Huali Chang, Jiming Zhang, Songhai Jia

**Affiliations:** 1 Henan Geological Museum, Zhengzhou, Henan, China; 2 Hokkaido University Museum, Hokkaido University, Sapporo, Japan; 3 Institute of Geology, Chinese Academy of Geological Sciences, Beijing, China; College of the Holy Cross, United States of America

## Abstract

Therizinosauria are an unusual group of theropod dinosaurs, found mostly in the Cretaceous deposits in Mongolia, China and western USA. The basal forms of this group are represented by incomplete or disarticulated material. Here, we report a nearly complete, articulated skeleton of a new basal therizinosaur from the Early Cretaceous Yixian Formation of Jianchang County, western part of Liaoning Province, which sheds light on our understanding of anatomy of basal therizinosaurs. This new dinosaur shows some typical therizinosaur features, such as neural spines of the anterior caudal vertebrae that possess anterior and posterior alae, a rectangular buttress on the ventrolateral side of the proximal end of metacarpal I, and appressed metatarsal shafts. Our phylogenetic analysis suggests that it is a basal therizinosaur (sister taxon to Therizinosauroidea) because it bears many basal therizinosaur characters in the dentition, pelvis and hind limbs. The new therizinosaur described here has unique tooth and jaw characters such as the offsetting of the tooth row by a shelf and dentary teeth with labially concave and lingually convex dentary teeth, similar to ornithopods and ceratopsians.

## Introduction

Therizinosauria are an unusual group of theropod dinosaurs, found mostly in the Cretaceous deposits in Mongolia, China, and western USA. Among twelve genera known so far, all of the primitive (non-therizinosaurid) therizinosaurs have been discovered in Asia except the basalmost form, *Falcarius utahensis*, which is from Utah [1], [2]. Three taxa have been reported from the Early Cretaceous in China; *Beipiaosaurus inexpectus* from the Yixian Formation (Barremian), *Alxasaurus elesitaiensis* from the Bayin-Gobi Formation (Aptian-Albian), and *Suzhousaurus megatherioides* from Xinminpu Group (Albian) [3]–[5].

*Beipiaosaurus inexpectus* was a sensational discovery because it was the most basal therizinosaur when it was described and it preserves primitive feathers. Despite the richness of vertebrate fossils from the Yixian Formation, this taxon is represented by only two skeletons (IVPP 11559 and STM31-1) [3], [6], [7] and is the only basal therizinosaur (non-therizinosaurid therizinosaur) known from the formation. Zanno [2] revised its diagnosis because the original diagnosis by Xu et al. [3] is based mainly on primitive features. The emended diagnosis by Zanno [2] is based on postcranial features (manual phalanx I-I, ischium, femur, pygostyle, and metacarpal I) because the skull preservation of IVPP 11559 is poor and the skull of STM31-1 is not described yet.

Kirkland et al. [8] described *Falcarius utahensis*, from the Lower Cretaceous Cedar Mountain Formation in Utah based on the most complete therizinosaur material known, represented by over 3000 disarticulated elements. After the description of this taxon, more intensive studies have been published on its braincase [9], forelimbs [10], and the remaining elements [11]. A recent phylogenetic analysis by Zanno [2] demonstrated that *Falcarius utahensis* is the most basal therizinosaur and is a sister taxon to the clade of Therizinosauroidea (the clade of *Beipiaosaurus inexpectus* and higher taxa). Zanno stated that there is a large morphological gap between *Falcarius utahensis* and *Beipiaosaurus inexpectus* and that *Beipiaosaurus inexpectus* is more derived than *Falcarius utahensis* mainly because of its dentition and pelvis.

**Figure 1:**
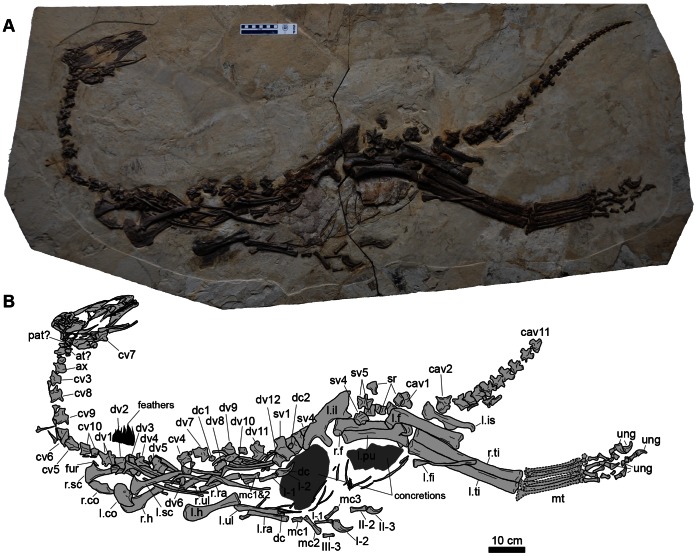
A photograph of the skeleton of *Jianchangosaurus yixianensis* gen. et sp. nov. (41HIII-0308A) (A) and line drawing (B). Abbreviations: at, atlas; ax, axis; cav, caudal vertebra; cv, cervical vertebra; co, coracoid; dc, distal carpal; dv, dorsal vertebra; l., left; f, femur; fi, fibula; fur, furcula; hu, humerus; il, ilium; is, ischium; pu, pubis; ra, radius; sc, scapula; ti, tibia; ul, ulna; mc, metacarpal; mt, metatarsal; mxf, maxillary fenestra; pat, proatlas; r., right; sr, sacral rib; sv, sacral vertebra; ung, ungual. All elements of the skeleton are preserved except the distal half of the caudal vertebrae. Dashed lines of metatarsals indicate areas that have been reconstructed. The middle portion of the neck, from the fourth to ninth cervical vertebrae, and the pedal phalanges have been repositioned. The rest of elements of this specimen are in the original position.

**Figure 2:**
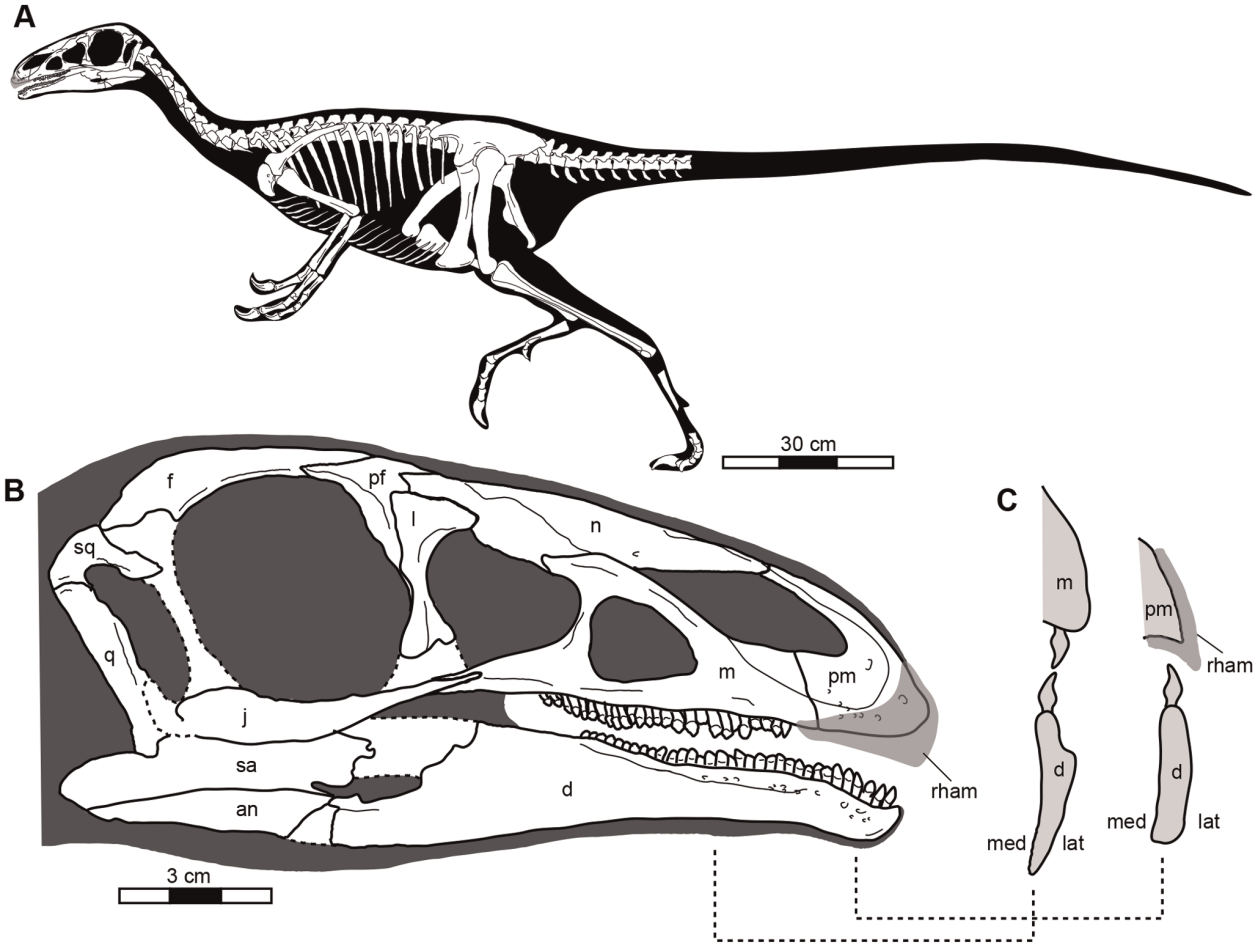
A, Reconstruction the skeleton of *Jianchangosaurus yixianensis* gen. et sp. nov. The hip height is approximately 1 meter. It is 1880 mm long from the skull to the eleventh caudal vertebra. B, Reconstruction of the skull. The ventral lacrimal, postorbital, posterior jugal, anterior edge of the quadrate, anterior surangular are missing. A rhamphotheca is reconstructed in grey based on edentulous area and a series of foramina. C, Cross section of the right upper and lower jaws, showing dental arrangement and rhamphotheca. Anterior portion (right) may have been used for plucking food by a rhamphotheca in the upper jaw and anterior dentary teeth with the normal dental morphology (convex labial and concave lingual surfaces). Posterior portion (left) shows the opposite dental morphology (concave labial and convex lingual surfaces) in the dentary, which allows the tips of the upper and lower teeth to abut each other. Abbreviations: an, angular; d, dentary; f, frontal; j, jugal; l, lacrimal; lat, lateral; m, maxilla; med, medial; n, nasal; pf, prefrontal; pm, premaxilla; q, quadrate; rham, rhamphotheca; sa, surangular; sq, squamosal.

A skeleton of a new basal therizinosaur (41HIII-0308A), discovered from the Early Cretaceous Yixian Formation of Jianchang County, in the western part of Liaoning Province, was purchased by the Henan Geological Museum in Henan Province, China (Figure 1). Although the skeleton appears to be well preserved, some bones were apparently repositioned during its preparation. The skull and most parts of its body, from the shoulder to tail, including many vertebrae (anterior cervicals [atlas to cervical 3], cervicodorsals [cervical 10 to dorsal 5], and posterior to dorsal 8 up to caudal 11) are in the original position. Some middle cervical vertebrae and the hind limb elements (metatarsals and pes) are repositioned. The similar size of all vertebrae, lack of overlap of elements, preservation of all vertebrae from atlas to caudal 11, and other elements in the original positions suggest that all bones belong to a single individual. It is the most complete articulated skeleton of any therizinosaur, missing only the distal tail (Figures 1A, B, 2A). Here, we describe this nearly complete new basal therizinosaur, which sheds light on understanding the anatomy and evolution of the group.

## Methods

A phylogenetic analysis was performed using TNT (Tree Analysis Using New Technology) v. 1.1 [12] and the data matrix of Zanno [2] with the addition of the new therizinosaur described here (Appendix 1 in File S1). Most parsimonious trees were obtained by heuristic search methods on 1000 replicates of Wagner trees with random addition sequences and subject to tree bisection-reconnection swapping methods holding 10 trees per replicate. As noted by Zanno [2], twenty characters (characters 27, 37, 40, 68, 76, 78, 97, 106, 113, 157, 163, 168, 253, 303, 308, 309, 310, 334, 342, and 345) were designated additive and two characters (characters 165 and 125) are excluded.

This study follows Zanno [2], Clark et al. [13], Zanno et al. [14] for the definitions of Therizinosauria, Therizinosauroidea, and Therizinosauridae, respectively. Therizinosauria is the most inclusive clade containing *Therizinosaurus cheloniformis* but not *Tyrannosaurus rex, Ornithomimus edmontonicus, Mononykus olecranus, Oviraptor philoceratops or Troodon formosus*. Therizinosauroidea is defined as the least inclusive clade containing *Beipiaosaurus inexpectus* and *Therizinosaurus cheloniformis*. Therizinosauridae is the least inclusive clade containing *Nothronychus*, *Segnosaurus galbinensis*, *Erlikosaurus andrewsi*, and *Therizinosaurus cheloniformis*.

### Nomenclatural Acts

The electronic edition of this article conforms to the requirements of the amended International Code of Zoological Nomenclature, and hence the new names contained herein are available under that Code from the electronic edition of this article. This published work and the nomenclatural acts it contains have been registered in ZooBank, the online registration system for the ICZN. The ZooBank LSIDs (Life Science Identifiers) can be resolved and the associated information viewed through any standard web browser by appending the LSID to the prefix "http://zoobank.org/". The LSID for this publication is: urn:lsid:zoobank.org:pub:2347B9A7-1C7A-4C0A-AC64-A45817E797AB. The electronic edition of this work was published in a journal with an ISSN, and has been archived and is available from the following digital repositories: PubMed Central, LOCKSS.

## Results

Systematic Paleontology

Theropoda Marsh 1881

Coelurosauria von Huene 1914

Therizinosauria Russell 1997

*Jianchangosaurus yixianensis* gen. et sp. nov. urn:lsid:zoobank.org:act:9F8AF9B5-B17A-4E1D-BCCE-D8EA05D2067E.

### Etymology

Jianchang: the county of Liaoning Province, China, where the specimen was found; saurus: lizard; yixian: referring to the formation which yielded this specimen.

### Holotype

41HIII-0308A (Henan Geological Museum), a nearly complete juvenile skeleton with skull (including mandibles) (Fig. 1).

### Horizon and Locality

Yixian Formation. Niujiaogou of Jianchang, Liaoning Province [15].

### Diagnosis

A basal therizinosaur, bearing the following unique combination of characters; 27 tightly packed maxillary teeth; dorsal border of the antorbital fenestra formed by maxilla, nasal, and lacrimal, with the majority of the border formed by the nasal; no participation of jugal in margin of antorbital fenestra; a short diastema in anterior tip of dentary; concave labial surface and convex lingual surface of dentary teeth (except six anterior teeth); lack of prominent hypapophyses in anterior dorsal vertebrae; anterior caudal centra with an oval cross section and articular facet as tall as wide; weakly curved manual unguals with weak flexor tubercles ventral to articular facet; shallow and elongated ilium; a ridge bounding cuppedicus fossa confluent with acetabular rim; extensive contact between pubic apron.

### Description

*Jianchangosaurus yixianensis*, represented by a nearly complete skeleton missing the distal tail, is a gracile dinosaur 1 meter in height at the hips and 2 meters in estimated length, thus shorter than *Falcarius utahensis* (Table 1 and Figures 1A, B, 2A).

**Table 1:** Measurements in millimeters of selected portions of *Jianchangosaurus yixianensis* gen. et sp. nov. (41HIII-0308A).

Measured portion	Length	Height
Skull	230	110
Hyoid	88.8	
Neck	440	
Body	510	
Tail*	550	
Total*	1880	

Asterisks indicates measurements of preserved portions.

**Figure 3:**
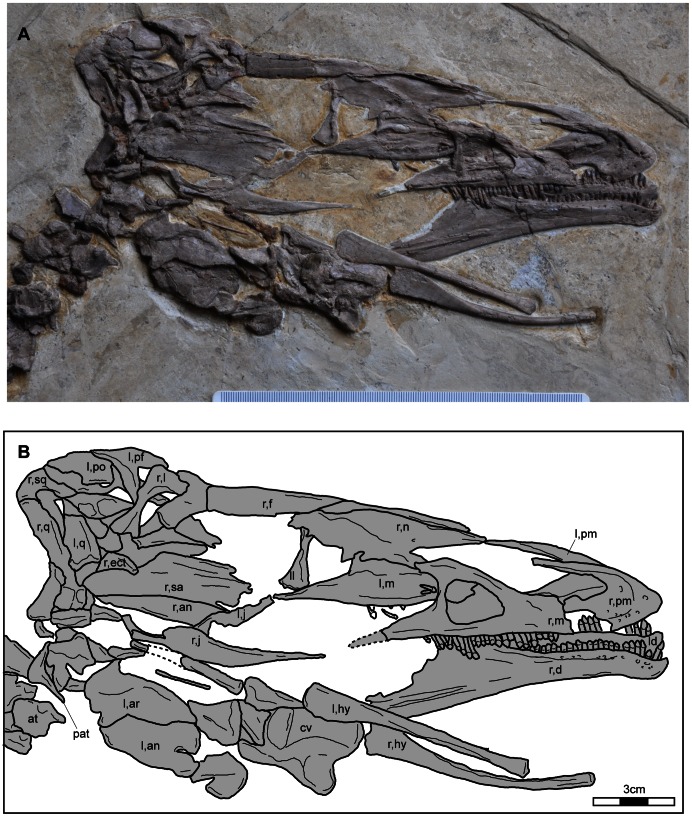
Photograph of the skull of *Jianchangosaurus yixianensis* gen. et sp. nov. (A) and line drawing (B). The right side of the skull is better exposed than the left side. The braincase and palatal bones are displaced. The seventh cervical vertebra is preserved next to the hyoids. Abbreviations: ect, ectopterygoid; hy, hyoids; mxf, maxillary fenestra; A, a large foramen at the base of the internarial bar of the premaxilla; B, a large foramen within the narial fossa of the premaxilla; C, a maxillary foramen above the eighth maxillary tooth; see captions for figures 1 and 2 for other abbreviations. Scale bars are 3 cm.

The right side of the skull is well exposed, but the braincase and palatal bones are displaced and the identity of these elements is unclear (Figure 3A, B). Because some skull elements are crushed or stacked on each other, it is difficult to identify these elements. The identified skull elements include both premaxillae, both maxillae, right frontal, both lacrimals, left prefrontal, left postorbital, both jugals, right squamosal, both quadrates, right ectopterygoid, both dentaries, left articular, and left angular. The skull is longer than the femur, unlike *Beipiaosaurus inexpectus*. The nares are large as in other therizinosaurs and extend near to the anterior edge of the antorbital fossa.

**Figure 4:**
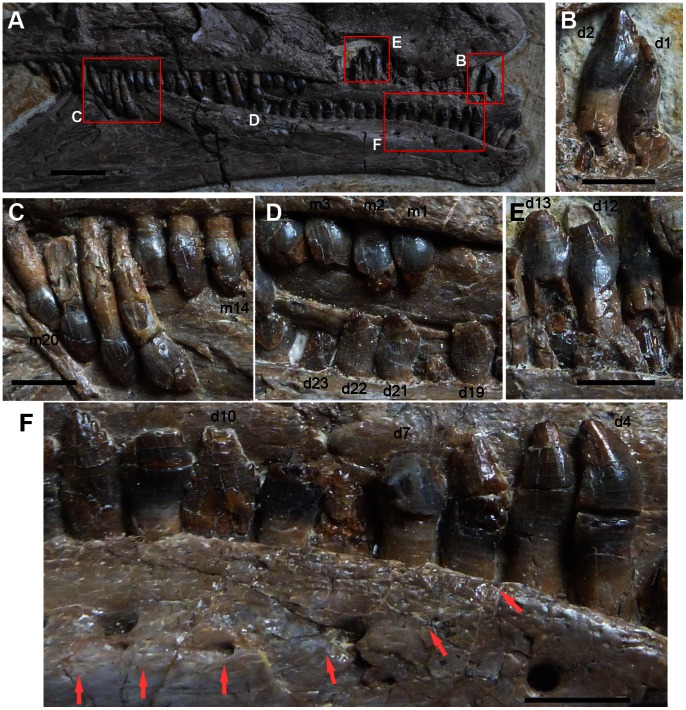
Dentition of *Jianchangosaurus yixianensis* gen. et sp. nov. A, Teeth in the upper and lower jaws. Labial surfaces of the right maxillary and dentary teeth and lingual surfaces of the left dentary teeth are exposed. The premaxilla and anterior tip of the dentary are edentulous. B, Anterior left dentary teeth (first and second) in lingual view, showing the convex lingual surface. A replacement tooth is exposed at the root of the second dentary tooth. C, Posterior right maxillary teeth (from fourteenth to twentieth) in labial view. The crown size diminishes posteriorly. D, Middle right maxillary and dentary teeth in labial view. The maxillary teeth exhibit the conventional dental morphology (convex labial surfaces), but the dentary teeth show the reversed dental morphology (concave labial surfaces). E, Middle left dentary teeth (from eleventh to thirteenth) in lingual view, showing the reversed dental morphology (convex lingual surfaces). F, Anterior left dentary teeth in labial view, showing a shelf (red arrows; starting between d6 and d7) and a shift from normal (d4 to d6; convex labial surface) to reversed (d7 and posterior; concave labial surface) teeth. Scale bars on A–F are 1 cm.

Among the basal therizinosaurs, only *Beipiaosaurus inexpectus* [7] preserves the premaxilla, but it has never been described. The best representative of the premaxilla among therizinosaurs is that of *Erlikosaurus andrewsi* [1], and that of *Jianchangosaurus yixianensis* is similar in general shape. The premaxilla (Figures 3A, B, 4A) is edentulous and its main body relative to the maxilla is much smaller than in *Erlikosaurus andrewsi* [1]. The premaxillary internarial bar is nearly vertical at its base and bends posteriorly. It extends across half of the external nares posterior to the premaxilla-maxilla contact. The dorsal surface of the bar is flat, and the cross-section of the bar is triangular. The maxillary process is short and has a pointed tip. In lateral view, the posterior edge of the main premaxillary body is concave for the maxillary contact. The curvature of this concavity is stronger than that of *Erlikosaurus andrewsi.* The outline of the narial fossa is large and occupies most of lateral surface of the main body of the premaxilla, unlike *Erlikosaurus andrewsi*. A large foramen is present on the lateral surface of the internarial bar (Figures, 3A, B, 4A). Another large foramen is present within the narial fossa, whereas three foramina are present in *Erlikosaurus andrewsi.* The foramen of the narial fossa perforates the premaxillary body. Its medial opening can be seen on the medial surface of the right premaxilla. In addition to these two foramina, a series of small neurovascular foramina is aligned along the ventral edge of the main body.

The anterior end of the anterior ramus of the maxilla is edentulous (Figures, 2B, 3A, B, 4A). The edentulous area is roughly 5 mm long. The anterior ramus has a smooth surface. It has one distinct maxillary neurovascular foramen above the eighth maxillary tooth and a groove posterior to the seventeenth tooth (Figure 3A, B) similar to *Erlikosaurus andrewsi* [13]. *Falcarius utahensis* has seven small maxillary foramina and lacks the large maxillary foramen [11]. The contact surface with premaxilla is exposed and shows a ridge and groove articulation. The anterior part of the antorbital fossa is well preserved. A large maxillary fenestra (Figures 2B, 3A, B), half the size of the antorbital fenestra, is present as in some troodontids (*Sinovenator changii*, *Saurornithoides mongoliensis* and *Zanabazar junior*) [16], [17] but is absent in *Erlikosaurus andrewsi*. The maxillary fenestra is as long as high as in *Sinovenator changii*, whereas that of derived troodontids is oval-shaped, long and low in lateral view [16]. The nasal process is short, unlike *Erlikosaurus andrewsi*, and extends posteriorly across one-fourth of the antorbital fenestra. The dorsal margin of the antorbital fossa (Figure 2B) is formed primarily by the nasal, which is unique to *Jianchangosaurus yixianensis*. The antorbital fenestra is positioned posterior to the narial opening and does not extend as far ventral, relative to the narial opening, as the antorbital fenestra of *Erlikosaurus andrewsi*. The jugal process is dorsoventrally high at the level of the interfenestral bar and tapers out posteriorly, where it contacts the jugal. The medial surface of the left maxilla is exposed and is smooth.

The nasal is long (Figure 3A, B), and extends from the level of the middle of narial opening to the lacrimal. The dorsal surface of the nasal is convex in cross section. The ventral border of the anterior one-third of the nasal is straight and borders the dorsoposterior margin of the narial opening (Figure 2B). The tip of the anterior end has a short overlap with the narial process of the premaxilla. In contrast, the overlap of the nasal and premaxilla is long in *Erlikosaurus andrewsi* [16]. The posterior portion of the nasal is transversely wide, as seen in *Falcarius utahensis* [11]. It is widest at the level of the nasal-maxilla contact. The posterior portion of the nasal possesses a short process, which fits onto the dorsal surface of the prefrontal. A small foramen is present near the nasal-maxilla contact.

Both lacrimals are preserved. The lateral surface of the right lacrimal and the medial surface of the left lacrimal are exposed (Figure 3A, B). The lacrimal contacts the large prefrontal and resembles an inverted L-shape (Figures 2A, B, 3A, B), rather than being T-shaped as in *Erlikosaurus andrewsi* [1], and is similar to that of ornithomimosaurs. A lacrimal recess is present on the anterior surface of the dorsal part of the lacrimal, which is much deeper than in *Erlikosaurus andrewsi*. It is unclear if any aperture is present in the recess on the anterior surface or the lacrimal foramen on the posterior surface of the dorsal region, due to crushing. A vertical lamina on the medial surface of the lacrimal extends from the posterior dorsal side to the anterior ventral side. The ventral portion of both lacrimals is not preserved.

The prefrontal is triangular-shaped in lateral and dorsal views (Figure 3A, B). The anterior edge of the dorsal portion is V-shaped for a contact with the lacrimal. The ventral process contacts the medial surface of the lacrimal and extends onto the ventral half of the lacrimal. In dorsal view, the contact with the frontal is nearly straight. Anteroposteriorly, the length of the dorsal portion of the prefrontal is slightly longer than that of the lacrimal. In dorsal view, the dorsal exposure of the prefrontal is larger than that of the lacrimal, unlike *Erlikosaurus andrewsi*, which has a prefontal that is smaller than lacrimal [1]. The prefrontal forms the anterodorsal rim of the orbit. The involvement of the prefrontal in the formation of the orbital rim is larger than in *Erlikosaurus andrewsi*.

The lacrimal, prefrontal, and squamosal have been displaced due to post-mortem deformation. They cover the anterior portion of the frontal, but the outline of the frontal can be traced (Figure 3A, B). The posterodorsal rim of the orbit is formed by the frontal (Figure 2B). The orbital rim is smooth as in *Falcarius utahensis* [11] but unlike *Erlikosaurus andrewsi* [1]. The frontal is widest at the frontal-postorbital contact and narrows anteriorly. Its dorsal surface is slightly concave and smooth. A brief contact between the lacrimal and frontal is seen in *Erlikosaurus andrewsi* [1], but there is no contact between these elements in *Jianchangosaurus yixianensis* because the posterior end of the frontal fits in the anterior border of the prefrontal. The contact with the postorbital is not preserved due to specimen deformation.

The postorbital is not well preserved. The squamosal is well preserved and has postorbital and quadratojugal processes (Figures 2B, 3A, B). The postorbital process is longer than the quadratojugal process. The postorbital process contacts the posterior edge of the postorbital.

The main body and anterior process of the jugal is preserved (Figure 3A, B). The anterior process of the jugal is flat transversely. The arrangement of the jugal-lacrimal contact is not clear because of poor preservation of the ventral part of the lacrimal; however there is a contact between these elements to form the anteroventral rim of the orbit. The anterior process of the jugal is long and tapers into a splint-like end. It fits on the lateral side of the maxilla but is not involved in the formation of the rim of the antorbital fossa (Figure 2A, B). In contrast, the anterior tip of the jugal of *Erlikosaurus andrewsi* overlaps the dorsolateral surface of the jugal process of the maxilla, and is involved in the antorbital fossa rim [1]. Because the posterior portion of the jugal is poorly preserved, the relationships with the postorbital and quadratojugal are unknown.

The quadratojugal is not preserved. The main body of the quadrate is preserved (Figure 3). It is straight in lateral view (Figure 2). There is no sign of pnumatization. There is a ridge on the lateral surface of the body along the posterior border of the infratemporal fenestra. The lateral mandibular condyle is exposed and is rounded.

The right mandible is well preserved except for the posterior end of the dentary (Figure 3A, B). The dentary is triangular in lateral view and has a down-turned symphyseal region anterior to the lateral shelf, posterior to the ninth dentary tooth, as in other therizinosaurs, except *Falcarius utahensis* [8], [11] (Figure 3A, B). The degree of curvature of the down-turned portion is less than any other taxon within this group (Figure 2A, B), except *Falcarius utahensis*. The symphyses are short, unfused and edentulous (Figure 4A). The edentulous region of *Erlikosaurus andrewsi* [1] is long, but it is short in *Jianchangosaurus yixianensis*. The dentary tooth row is inset from the lateral main surface of the dentary by a shelf (Figures 4A, F). The shelf extends from the fifth to at least the twenty-sixth tooth position and is wide between the tenth and twenty-second dentary teeth. A series of neurovascular foramina is present along the shelf (Figures 3A, B, 4A). The contact with the surangular at the posterior end of the dentary suggests that the dentary process of the surangular is dorsoventrally tall. The angular process of the dentary is tall dorsoventrally and forms the ventral border of the mandibular fenestra. The fenestra is one-third of the angular length (Figure 2B), which is smaller than in *Erlikosaurus* (half of the angular).

The surangular is tall as in *Erlikosaurus* (Figures 2B, 3A, B). Its lateral surface is smooth and lacks a foramen, similar to *Erlikosaurus*. A ridge is present along the dorsal edge of the surangular and lateral to the glenoid. The retroarticular process is robust and projects posteriorly (Figure 2B), as seen in *Erlikosaurus* [1]. The straight surangular-angular suture extends from the posteroventral corner of the mandibular fenestra to the posteroventral edge of the retroarticular process. The angular is shorter than the surangular because its dentary process is short and meets the dentary close to the posteroventral corner of the mandibular fenestra. The paired hyoids are rod-like anteriorly and plate-like posteriorly. The hyoids are about half of the length of the skull (Figure 3A, B).

*Jianchangosaurus yixianensis* possesses 27 maxillary teeth. The precise number of the dentary teeth is not clear because the seventh and eighth maxillary teeth cover the dentary teeth posterior to the twenty-fifth position: however, based on the space covered by the seventh and eighth maxillary teeth, the number of dentary teeth likely ranges between 25 and 28 (Figures 3A, B, 4A, C). The number of maxillary teeth is similar to *Falcarius utahensis* (25) [8], [11], *Segnosaurus galbinensis* (24) [19], and *Erlikosaurus andrewsi* (31) [16]. All teeth are small, lanceolate, constricted at the crown base with no replacement gaps, and have long cylindrical roots. *Jianchangosaurus yixianensis* does hot have enlarged rostral teeth as seen in *Falcarius utahensis* and *Incisivosaurus gauthieri* [8], [11], [19]. The maxillary teeth diminish slightly in size from mesial to distal as in other therizinosaurs but are aligned along the lateral edge of the maxilla, whereas the maxillary teeth are inset from lateral edge of the maxilla in *Erlikosaurus andrewsi* [1], [13]. The labial surfaces of all preserved maxillary teeth are hemispherical. In the dentary, the six anterior tooth crowns have a convex labial surface, but, surprisingly, posterior to the seventh tooth they have a concave labial surface (Figure 4E), and the lingual surface is convex (Figure 4D, F). This crown shape is not due to its preservation, even though it appears that some teeth are pressed against the ventral margin of the dentary. The crown shapes of the dentary teeth from the tenth to twenty-first dentary tooth positions are consistent, and three teeth (fifteenth, eighteenth, and twenty-first) are not pressed against the dentary. The lingual surfaces of middle dentary teeth are exposed (Figure 4E) and their bases are distinctly convex and rounded as seen in the labial surface of posterior maxillary teeth, but the labial surfaces of the middle dentary teeth near the crown base are slightly concave. Denticles along the margin of maxillary and dentary tooth crowns number three per millimeter, similar to *Beipiaosaurus* [3], but more than in *Falcarius utahensis* [8] and *Erlikosaurus andrewsi* [1]. The teeth of *Jianchangosaurus yixianensis* share a plesiomorphic feature with *Falcarius utahensis*, namely consistent denticle size, but differ from *Erlikosaurus andrewsi* and *Falcarius utahensis* in the teeth being closely packed in the anterior maxilla. *Jianchangosaurus* also differs from *Falcarius utahensis* in having the anterior dentary teeth similar in size to the other dentary teeth.

**Figure 5:**
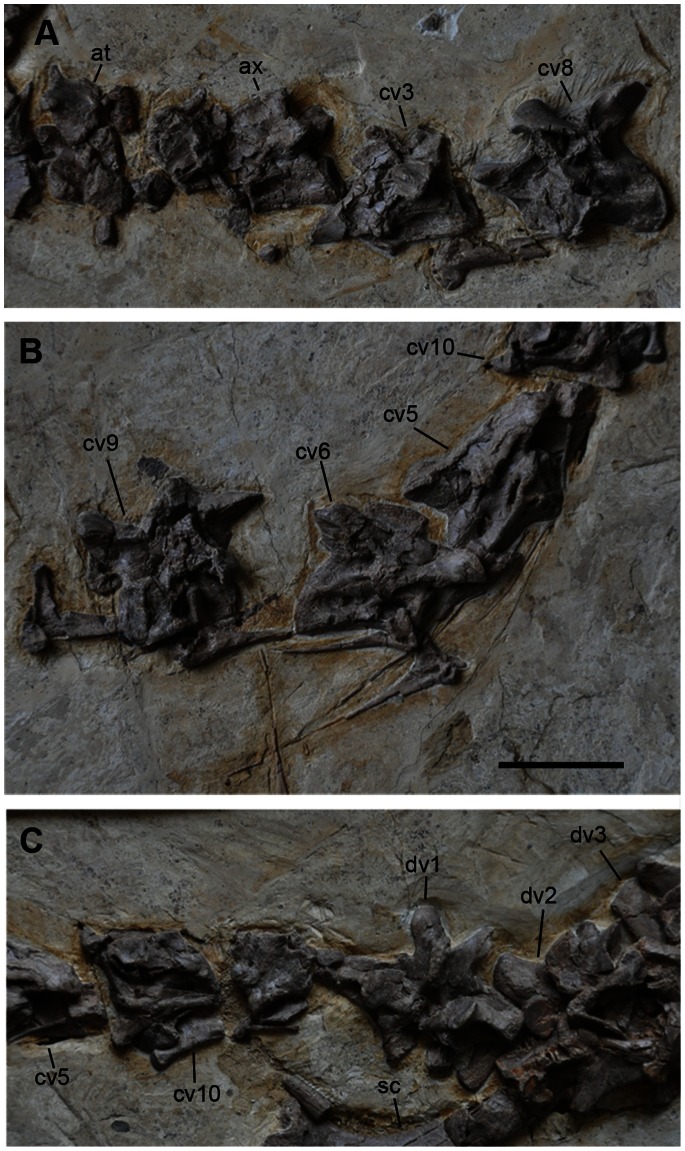
Cervical and anterior dorsal vertebrae of *Jianchangosaurus yixianensis* gen. et sp. nov. A, Atlas, axis and cervical vertebrae 3 and 8 in left lateral view. Cervical 8 is repositioned, but others are in the original position. B, Cervical vertebrae 5 and 6 in right lateral view and cervical vertebra 9 in left lateral view. Cervical 5 and 6 are articulated and repositioned on the opposite side. Cervical 9 is also repositioned. C, Cervical vertebra 10 and dorsal vertebrae 1–3. All are in the original position. See caption of Figure 1 for abbreviations. Scale bar is 5 cm for A–C.

**Figure 6:**
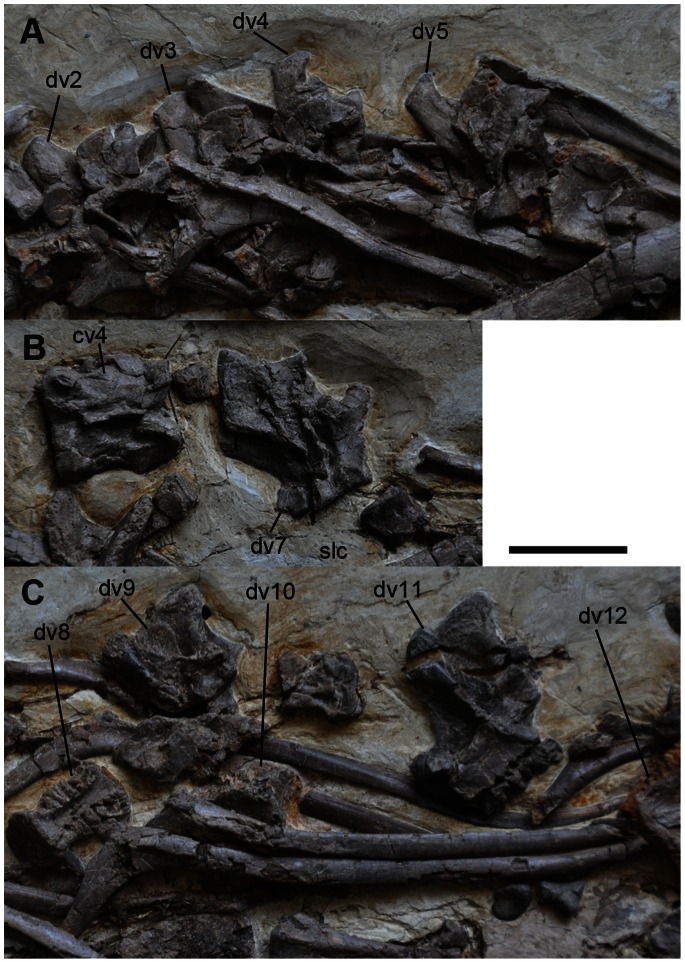
Dorsal vertebrae and cervical vertebra 4 of *Jianchangosaurus yixianensis* gen. et sp. nov. All neurocentral sutures are unfused. A, Articulated dorsal vertebrae 2–5 and ribs in left lateral view. B, Cervical vertebra 4 in dorsal view and dorsal vertebra 7 in left lateral view. A partial semilunate carpal can be observed next to dorsal vertebra 7. C, Semi-articulated dorsal vertebrae 8–12, likely displaced post-mortem. Abbreviations: slc, semilunate carpal; see caption of Figure 1 for other abbreviations. Scale bar is 5 cm for A–C.

**Figure 7:**
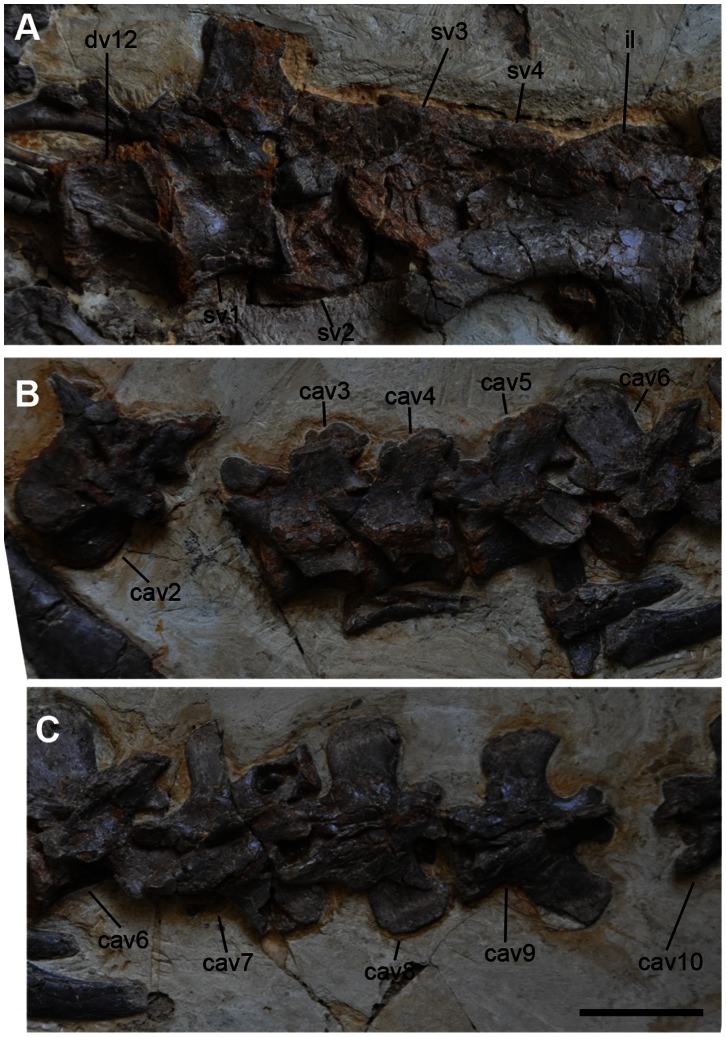
Sacral and caudal vertebrae of *Jianchangosaurus yixianensis* gen. et sp. nov. A, Articulated vertebrae from dorsal 12 to sacral 4. The lateral side of the first and second sacral vertebrae is exposed. The sacral centra are unfused, but the neutral spines of the third and fourth sacral vertebrae are fused into a single plate. B, Articulated caudal vertebrae (from the second to sixth). The distal tips of the neural spines have anterior and posterior alae. C, Articulate caudal vertebrae (from sixth to tenth) in dorsal view. See caption of Figure 1 for abbreviations. Scale bar is 5 cm for A–C.

All vertebrae up to the eleventh caudal vertebra are preserved (Figures 5, 6, and 7). The fourth cervical vertebra is misplaced between the fifth and seventh dorsal vertebrae (Figure 6B). The fifth and sixth cervical vertebrae appear to be close to their original positions, but they are oriented in the wrong direction and they rest on the wrong side (Figure 5B). The seventh cervical vertebra lies near the skull, but this is because of post-mortem transportation before burial (Figure 3B). The eighth and ninth cervical vertebrae are displaced next to the third cervical vertebra (Figure 5A, B). The rest of the caudal vertebrae are missing (Figures 1A, B, 5A–C, 6A–C, and 7A–C). The neurocentral sutures are fused in all caudal vertebrae, but open in cervical and dorsal vertebrae. Cervical ribs are also unfused, suggesting that the specimen is a juvenile [20].

All ten cervical centra are amphicoelous and highly pneumatized, with a pair of foramina in the mid-cervicals and a single foramen in the rest of the cervicals (Figure 5A–C). The lateral surface of the left atlas neural arch is exposed. Its postzygapophysis is short and has a rounded posterior end. The axis has a low neural spine with a straight dorsal border. The postzygapophyses are larger than the prezygapophyses and extend posterior to the neural spine. The epipophyses on the dorsal surface of the prezygapophyses are prominent. The postzygapophyseal articular surfaces face posteroventrally and are rounded. The axial centrum has a large pneumatic fossa on its lateral surface. The third to sixth cervical vertebrae are low in height and the cervical vertebrae become longer posteriorly (Table 2). The neural arches of the middle to posterior cervicals are X-shaped in dorsal view (Figure 6B), similar to other therizinosaurs and oviraptorosaurs [21]. The neural spines of the middle to posterior cervicals (from the fourth to tenth) are low. The prezygapophyses of the fourth and more posterior cervical vertebrae are long and extend beyond to the anterior edge of the centrum. The prezygapophyses are robust and have oval-shaped articular surfaces. The postzygapophyses of the third and fourth cervical vertebrae have epipophyses. These are short and connected by a lamina so that the posterior border of the neural arch in dorsal view is straight. The postzygapophyses of the posterior cervical vertebrae (from fifth to tenth) are separated. The intervertebral articular surfaces of the fifth to seventh vertebrae are inclined anteriorly, whereas the rest of cervical centra are nearly vertical. The lateral surfaces of the third, fifth, sixth, eighth, and ninth vertebral centra are well exposed. The anterior lateral surface of the third centrum exhibits a large pneumatic fossa, similar to the axis (Figure 5A). There are paired large pneumatic fossae on the fifth, sixth, and eighth cervical centra (Figure 5A, B).

**Table 2:** Measurements in millimeters of vertebrae of *Jianchangosaurus yixianensis* gen. et sp. nov.

Element	Length, maximum	Height, maximum	
Axis	25.2	29.6	
Cervical vertebra 3	42.5	32.7	
4	33.2	−	
5	42.6	26.7	
6	47.4	32.7	
7	45.5	−	
8	45.9	38.1	
9	45.5	40.1	
10	47.2	−	
	**Length, neural arch**	**Length, centrum**	**Height, maximum**
Dorsal vertebra 1	40.8	−	−
2	−	23	49.8
3	−	−	50
4	−	−	46.5
5	37.6	25.4	46.3
6	−	−	−
7	40.5	−	−
8	−	27.4	−
9	−	27.3	−
10	−	−	−
11	39.8	−	−
12	−	26.9	−
	**Length, neural spine**	**Length centrum**	
Sacral vertebra 1	20.7	29.9	
2	19.6	−	
3	20.1	−	
4	19.8	30.9	
5	−	28.6	
	**Length, neural arch**	**Length centrum**	
Caudal vertebra 1	−	26.6	
2	−	18.2	
3	35.4	23.2	
4	38.2	24.2	
5	36.4	25.5	
6	31.8	−	
7	38.5	−	
8	38.1	−	
9	33.9	−	
10	38.9	−	
11	−	−	

The dorsal surface of the first dorsal vertebra is exposed. It has a longer neural spine than any of the cervical vertebrae (Table 2). The transverse processes project posterolaterally in dorsal view. The outline of the articular surfaces of the prezygapophyses is circular in shape. Most of the anterior dorsal vertebrae, from the second to fifth, are covered by dorsal ribs. The neural spines of the dorsal vertebrae are slightly inclined posteriorly and become progressively taller and longer posteriorly (Table 2). In lateral view, the dorsal edges of the neural spines of the anterior dorsal vertebrae are rounded, but they are square-shaped in middle to posterior dorsal vertebrae. The transverse processes increase in length posteriorly and their tips are expanded. The prezygapophyses and postzygapophyses of the dorsal vertebrae are much shorter than those of the posterior cervical vertebrae. In lateral view, the prezygapophyses are at the same level as the anterior edge of the centrum, whereas the postzygapophyses extend posterior to the vertebral centrum. All dorsal centra are spool-shaped and apneumatic (Figure 6A–C).

Five sacral vertebrae are present in *Jianchangosaurus yixianensis,* similar to basal therizinosaurs (Figure 7A). The fourth and fifth sacral vertebrae are covered by the ilium, except for their neural spines and ventral surfaces. The first sacral vertebra is better preserved than the other sacral vertebrae, and the second and third sacral vertebrae are heavily crushed. All of the sacral centra are unfused. The neural spines of the third and fourth sacral vertebrae are fused. The first sacral neural spine has a straight dorsal and a vertical anterior edge in lateral view. The postzygapophyses of the first sacral vertebra are much smaller than those of the dorsal vertebrae. The centra of the first and second sacral vertebrae are apneumatic, unlike *Nothronychus* or oviraptorosaurs. A ventral groove is present on the ventral surface of the first sacral centrum.

The neural spines of the caudal vertebrae are tilted posteriorly and their distal ends are separated into anterior and posterior alae, similar to other therizinosaurs (Figure 7B, C) [4]. The prezygapophyses and postzygapophyses are short and have circular-shaped articular surfaces. The transverse processes project laterally in the first eight caudal vertebrae, and posterolaterally in more posterior caudal vertebrae. The centra are apneumatic and spool-shaped, and their interarticular surfaces are as high as they are wide.

**Figure 8:**
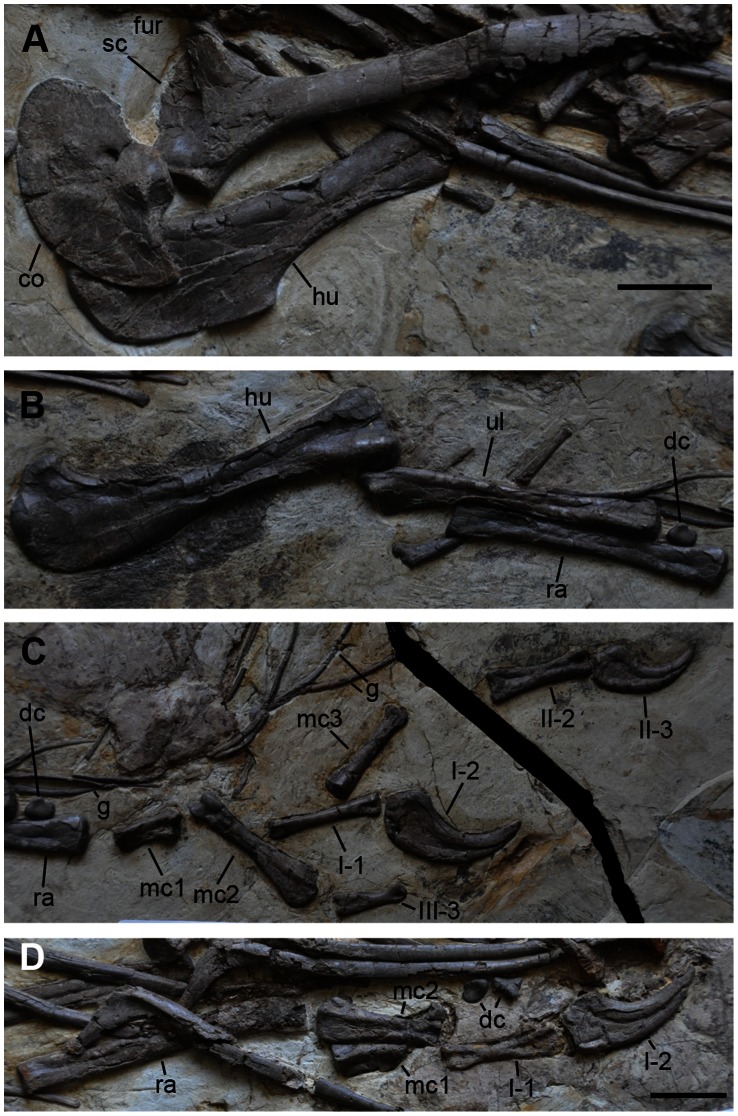
Pectoral girdle and forelimbs of *Jianchangosaurus yixianensis* gen. et sp. nov. A, Left pectoral girdle in lateral view and right humerus in ventral view. The scapula and coracoid are unfused. Scapular blade is straight and narrow. The coracoid is semicircular in shape. B, Left humerus, ulna, radius, and distal carpal (radiale). The forelimb is gracile. C, Disarticulated left metacarpals and manual digits. All metacarpals are preserved but some manual phalanges (II-1, III-1, III-2, and III-4) are missing. D, Right ulna, distal carpals (left, radiale; right, unidentified distal carpal), metacarpals, and manual phalanges. Metacarpal I shows a rectangular buttress for a contact with metacarpal II. Abbreviations: fur, furcula; g, gastralia; see caption of Figure 1 for other abbreviations. Scale bars are 5 cm. The scale bar on D is for panels B–D.

The ribs of the fifth, sixth, and ninth cervical vertebrae are well preserved. The cervical ribs are longer than their corresponding vertebral centra. The vertically oriented tubercula of the cervical ribs are longer than the horizontally oriented capitula. The medial surface of the cervical ribs is excavated by a pair of fossae. The posterior processes of the cervical ribs are thin and splint-like. The dorsal ribs are long and slender. The shafts of the dorsal ribs are curved anteriorly. The distal tips of the anterior dorsal ribs are expanded. At least sixteen splint-like gastralia are preserved (Figure 1A, B, 8B, C).

The scapula and coracoid are unfused, similar to basal therizinosaurs, which may be an ontogenetic feature in *Jianchangosaurus yixianensis* (Figure 8A). The right scapula is better exposed than the left scapula. The right scapula is nearly complete, but the posterior edge of the right scapular blade is damaged. The glenoid faces posteriorly. The acromion of the scapula is less developed than in *Falcarius utahensis* [10], and its margin is continuous with the scapular blade. The supraglenoid buttress of the scapula is larger than the infraglenoid buttress of the coracoid. The scapular blade is straight and its width is nearly constant from the base (14.8 mm) to the dorsal end (15.0 mm). Slight expansion of the blade is present in *Falcarius utahensis*, *Alxasaurus elesitaiensis*, and *Neimongosaurus yangi* [4], [10], [22], and a reduction of blade width is seen in *Therizinosaurus cheloniformis* [23].

The coracoid has a semi-circular outline with a long posterior process, similar to ornithomimosaurs (Figure 8A) [24]. The coracoid is much longer than it is high due to its long posterior process, unlike *Falcarius utahensis Neimongosaurus yangi, Segnosaurus galbinensis*, and *Suzhousaurus megatherioides*, which have a short posterior process [5], [10], [18], [22]. The dorsal border of the posterior process of *Jianchangosaurus yixianensis* is perpendicular to the main axis of the scapulocoracoid, unlike other therizinosaurs which exhibit an angle of approximately 45 degrees. The biceps tubercle is small and is positioned close to the base of the posterior process. The coracoid foramen is located near the scapula-coracoid suture. The distal tip of the furcula is poorly preserved, close to the left scapula (Figure 8A).

The forelimbs and hind limbs of *Jianchangosaurus yixianensis* are gracile similar to basal therizinosaurs (Figure 1A, B, 8A–D). The humerus is shorter than the scapula (Table 3). Both ends of the humerus are moderately expanded as in basal therizinosaurs. The deltopectoral crest extends along the proximal one-third of the shaft and its tip bends ventrally. The humeral head slightly protrudes posteriorly. The internal tuberosity of the humerus is large and is separated from the humeral head by a depression on the posterior surface, similar to *Falcarius utahensis* [10]. The humeral shaft is straight in ventral view, and the shaft diameter is narrowest at mid-length. The entepicondyle is reduced, unlike other therizinosaurs. The distal condyles are separated by a shallow groove on the posterior surface.

**Table 3:** Measurements in millimeters of appendicular elements of *Jianchangosaurus yixianensis* gen. et sp. nov.

Element	Length	Width		
Scapula, left	170.8	−		
Coracoid, left	75.5	−		
Humerus, left	158.5	50.1		
Ulna, left	124.3	20.8		
Radius, left	112	17.1		
Ulnare, left	11.4			
Intermedium, left	11.7			
Mc1, left	28.3			
Mc2, left	61			
Mc3, left	43.9			
I-1, left	46.6			
I-2, left	54.7			
III-3, left	29.7			
II-2, left	40.7			
II-3, left	45.4			
	**Total length**	**Height**		
Ilium, left	202.9	39.4		
	Length	Boot length		
Pubis, left	177.8	75.2		
Ischium, left	148.2			
	**Length**	**Width, proximal**	**Width, distal**	**Circumference**
Femur, left	206.6	59.6	42	65
Tibia, left	316	39.2	32.2	−
Fibula, left	167.1	20.3	−	−
Metatarsal III	171	−	−	−

The ulna is 78% of the humerus length (Table 3), which is close to the ratio observed in *Falcarius utahensis* (77%). Its proximal end is triangular in cross-section and has a less developed olecranon process compared to other therizinosaurs. The ulna is straight as in therizinosaurids (*Nothronychus*, *Erliansaurus*, and *Therizinosaurus*), but unlike *Falcarius utahensis* [10]. Its distal end is semicircular. The radius is straight and lacks the biceps tubercle, similar to *Falcarius utahensis*. Its shaft is slightly thinner than the ulnar shaft. The distal end of the radius is wider than its proximal end.

Four distal carpals (semilunate carpal, two radialia, and an unidentified carpal) are preserved. Half of the semilunate distal carpal is preserved and has been displaced post-mortem. It is now positioned between the seventh and eighth dorsal vertebrae (Figure 6B). Its proximal surface is exposed and shows a trochlear groove, similar to *Falcarius utahensis* [10]. A radiale, placed next to the left ulna and radius, is circular in shape and exhibits a slightly depressed surface (Figure 8C), which is similar to that of *Falcarius utahensis* [10]. Two other distal carpals are placed below the dorsal ribs near the right manual digits (Figure 8D). One of these distal carpals is a radiale, and the other is triangular in shape and is similar to an unidentified distal carpal of *Falcarius utahensis*.

All metacarpals, except the right metacarpal III, are preserved (Figure 8C, D). Metacarpal I is approximately half the length of metacarpal II (Table 3). Proximal metacarpal I has a rectangular buttress for contact with metacarpal II, and metacarpals I and II are stouter than metacarpal III, similar to other therizinosaurs. The medial distal condyle of metacarpal I is larger than its lateral condyle. Metacarpal II is straight. Its proximal end is rectangular-shaped in proximal view. The distal ends of metacarpals II and III have well-developed condyles. Metacarpal III has a contact surface for metacarpal II on the medial surface, which extends one-third of its length. The left hand preserves more phalanges than the right side (I-1, I-2, II-2, II-3, III-3 on left; I-1 and I-2 on right). Phalanx I-1 is the longest of the preserved phalanges. It is shorter than metacarpal II (Table 3). Its distal end is expanded for the articulation with the ungual and lacks a collateral ligament fossa. The other penultimate phalanges (II-2 and III-3) are similar to phalanx I-1, but the lateral surface of phalanx III-3 has a deep and circular collateral ligament fossa. The unguals are curved and transversely narrow. Ungual I-2 is larger, longer and more curved than ungual II-3. The height of the articular surface for penultimate phalanx I-1 on ungual I-2 is less than half of that of the proximal end. The proximal end lacks the dorsal lip as in basal therizinosaurs. The flexor tubercles of all unguals are rounded. A groove on the medial surface extends onto the dorsal surface of the ungual at its tip (Figure 8C, D).

**Figure 9:**
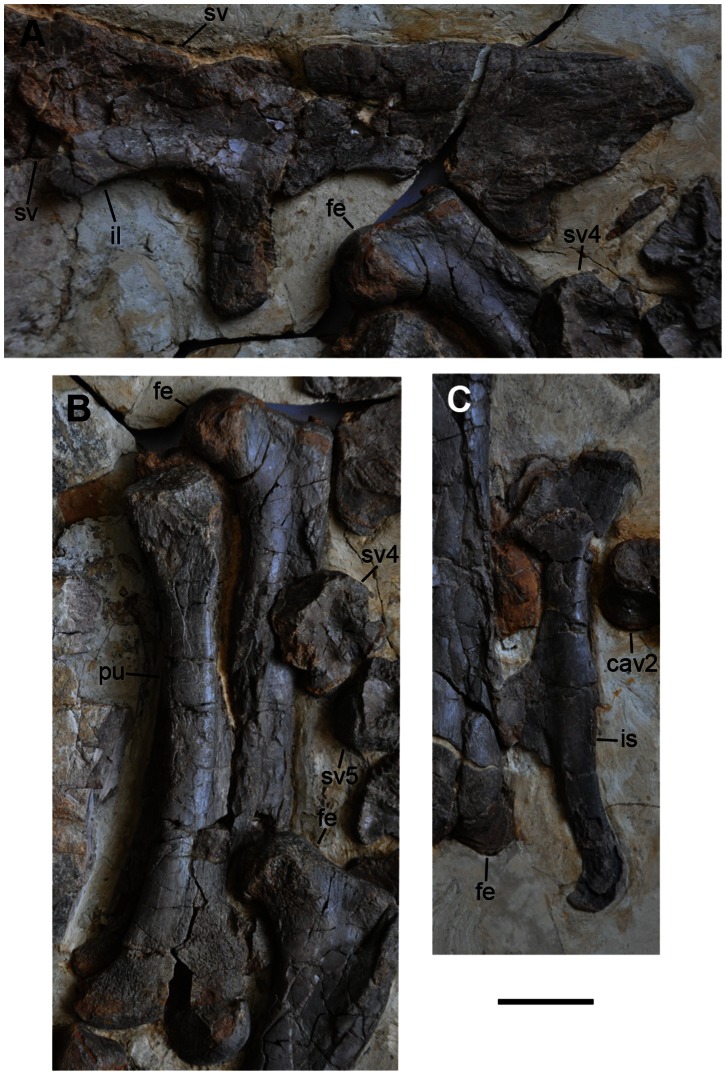
Pelvis and femora of *Jianchangosaurus yixianensis* gen. et sp. nov. A, Left ilium in lateral view. The ilium is low and dorsal edge is nearly horizontal in lateral view. B, Left pubis in lateral view and right femur in posterior view. The fourth sacral centrum is preserved next to the right femur. C, Left ischium in lateral view. A triangular obturator process is positioned at the mid-length of the element. See caption of Figure 1 for abbreviations. Scale bar is 5 cm for A–C.

The pelvis shows primitive features (Figure 9A). The ilium is low and its dorsal edge is nearly horizontal in lateral view, with a shallow preacetabular process and deep postacetabular process like many other coelurosaurian theropods, but unlike any other therizinosaurs with a dorsoventrally deep preacetabular blade (alti-iliac condition). Although the alti-iliac condition is absent in *Jianchangosaurus*, the ventral border of the preacetabular blade is higher than the dorsal margin of the acetabulum, similar to other therizinosaurs [11]. The preacetabular process gradually becomes lower anteriorly, its anterior tip is rounded and exhibits a slight anteroventral projection. The anteroventral projection of the preacetabular blade is much more developed than in other therizinosaurs (*Falcarius utahensis, Beipiaosaurus inexpectus*, *Segnosaurus galbinensis*, *Nothronychus graffami*) [2], [6], [11], [14]. The brevis shelf is not well developed. The outline of the postacetabular process is triangular-shaped and its tip points posteriorly. The pubic peduncle is long and its anteroposterior length remains uniform to its ventral end. The anteroposterior length of the pubic peduncle is much shorter than in *Falcarius utahensis* [11]. The acetabulum is large and lacks a supra-acetabular crest. The cone-shaped ischiac peduncle is blunt and short.

The pubis projects anteroventrally and does not display the opisthopubic condition. It is shorter than the ilium, unlike *Falcarius utahensis*. The iliac peduncle is reduced as in other therizinosaurs except *Falcarius utahensis* [11]. The ischiac peduncle is covered by the femur. Its shaft is straight in lateral view, in contrast to *Falcarius utahensis*, which has a slightly sigmoid shaft [11]. The apron extends across at least the lower half of the element. It has a shallow boot with a short anterior process and long posterior process similar to *Falcarius utahensis*, although the anterior extension in *Falcarius utahensis* is greater than that of *Jianchangosaurus*. The ventral border of the pubic boot is weakly concave.

The pubis is 20% longer than the ischium, unlike *Falcarius utahensis* which has a pubis that is 50% longer [11] (Table 3). This maybe a derived condition in *Jianchangosaurus*, because the length of the ischium of derived therizinosaurs is nearly equal to length of the pubis. The proximal two-thirds of the ischium are straight, and its distal third is sigmoid-shaped in lateral view. The ischiac shaft of *Falcarius utahensis* is concave posteriorly, similar to oviraptorosaurs. The iliac and pubic peduncles are short. The dorsoventral length of the pubis-ischium contact is longer than the anteroposterior length of the ilium-ischium contact. A triangular-shaped obturator process is positioned at the mid-length of the element, in contrast to other therizinosaurs, in which this process is located in the distal half of ischium. Its distal end is slightly expanded, and its tip projects anteroventrally in lateral view.

**Figure 10:**
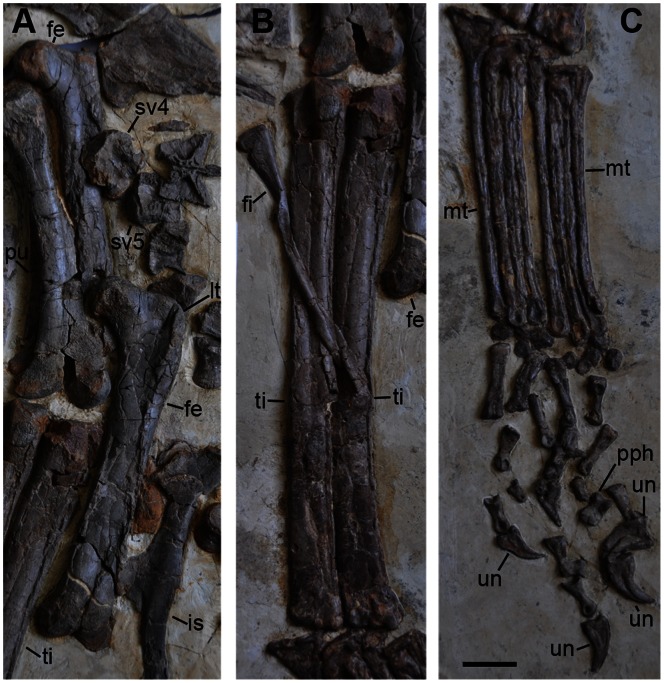
Hind limbs of *Jianchangosaurus yixianensis* gen. et sp. nov. A, Right (above) and left (bottom) femora in posterior and anterior views, respectively. B, Both tibiae and right fibula. The distal half of the fibula is missing. C, Metatarsals and pedal unguals. The metatarsals are covered in part by thick coatings, and the distal half of most metatarsals may not be real (reconstructed). The specific identification of most phalanges is not possible because most are crushed. Five bones are identified as pedal phalanges and have been labeled. Abbreviations: lt, lesser trochanter; pph, pedal phalanx; see caption of Figure 1 for abbreviations. Scale bar is 3 cm for A–C.

The femur is straight and has a cylindrical-shaped lesser trochanter, separated from the greater trochanter by a narrow cleft (Figure 10A). The neck of the head of the femur is weakly constricted. The axis of the femoral head is nearly perpendicular to the main axis of the femur. The fourth trochanter is not visible. The condyles at the distal end are separated by a sulcus. The tibia is 1.5 times longer than the femur, which is the highest ratio known in therizinosaurs, and suggests possible cursoriality in basal therizinosaurs (Figure 10A, B). Only the posterior surface of the tibia is exposed. The medial posterior process is well developed proximally and is separated from the laterally directed lateral posterior process. The fibula is much narrower than the tibia. Its proximal end is slightly expanded and the shaft width is one-third that of the proximal end. The astragalus and calcaneum are not exposed. Most of metatarsals of *Jianchangosaurus* appear to be reconstructed. The distal half of most metatarsals may not be real, although this cannot be confirmed because these bones are covered in part by thick coatings. However, some shafts are original, and they are rounded in cross-section and not appressed. The pedal phalanges are original but they are randomly displaced (Fig. 10C).

The pes of *Jianchangosaurus* is disarticulated. Identification of most phalanges is not possible because most are crushed and some may be from the manus. Five bones can be identified as pedal phalanges with certainty (Figure 10C). Four pedal unguals with poorly developed flexor tubercles are preserved. Two unguals are large and have deep lateral grooves. Two additional small unguals lack lateral grooves (Figure 10C). All of the pedal unguals are recurved and strongly compressed transversely, similar to *Erlikosaurus.* The pedal unguals are smaller than the manual unguals. The other phalanx (pph in Figure 10C) is similar to the penultimate phalanx of the digit IV in *Falcarius utahensis* [11]. It is wider than high and its distal condyles are separated by a sulcus.

**Figure 11:**
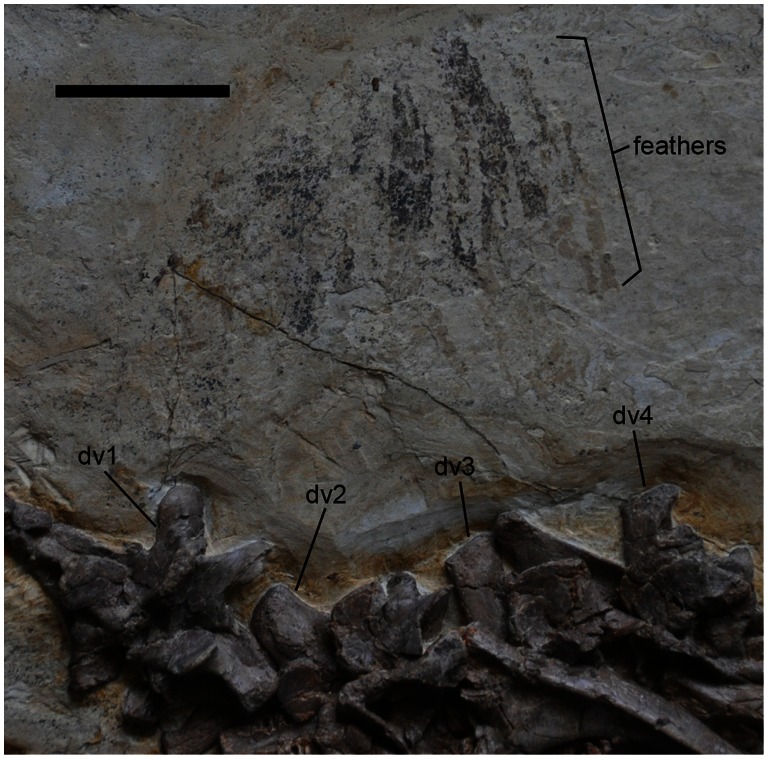
Feathers of *Jianchangosaurus yixianensis* gen. et sp. nov. The distal halves of the feathers are preserved as carbonized impressions, dorsal to the first to fourth dorsal vertebrae. These features are similar to the elongated broad filamentous feathers along the neck of *Beipiaosaurus*. See caption of Figure 1 for abbreviations. Scale bar is 5 cm.

Feathers are preserved as dark carbonized impressions dorsal to the first to third dorsal vertebrae (Figure 11). Because the bases of these feathers were destroyed during preparation, the length of feathers was estimated (10 cm) by measuring the distance from the tip of the feathers to the dorsal edge of the vertebrae. They are composed of wide and unbranched feathers. Their width varies between 2 to 3 mm. The length, width and unbranched structure of the feathers are similar to the elongated broad filamentous feathers (EBFF) along the neck of *Beipiaosaurus*, which was collected from the same formation at the same town, Jianchang. The presence of EBFF suggests that these feathers might have been used for visual display [7]. EBFF are different from the filamentous feathers (stage 1) seen in non-avian theropods [25], [26]. The elongated broad filamentous feathers along the neck of *Jianchangosaurus* are oriented nearly perpendicular to the long axis of the dorsal vertebral column, which is different from *Beipiaosaurus* (45 degrees), but may be due to preservation.

Two concretions are present in the body cavity, and can be distinguished from the surrounding matrix as bleached whitish colored aggregations. The anterior one is positioned near the middle dorsal vertebrae, and the posterior one is at the pelvis. No bony elements are present on the surface of the concretions.

### Phylogenetic Analysis

**Figure 12:**
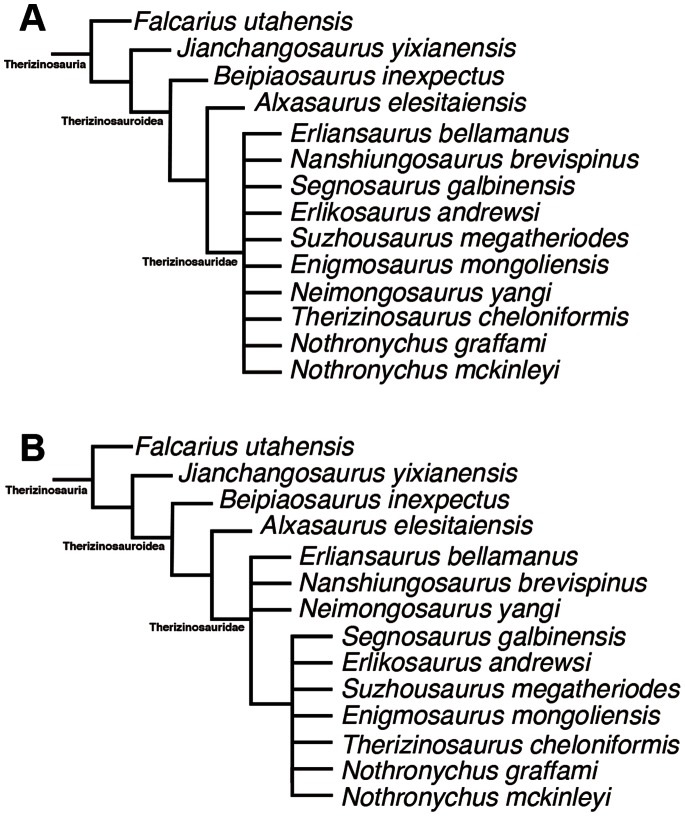
Strict consensus (A) and 50% Majority-rule consensus (B) of the forty most parsimonious trees recovered in this study. See Appendix 2 in File S1 for synapomorphies at each node.

The phylogenetic analysis resulted in forty most parsimonious trees with tree lengths of 1232. A strict consensus tree of the most parsimonious trees places *Jianchangosaurus yixianensis* as a basal therizinosaur (Figure 12A). The majority-rule consensus tree shows better resolution for the relationships of derived therizinosaurs (Figure 12B). Addition of *Jianchangosaurus yixianensis* to the analysis by Zanno [2] changes character distributions of basal therizinosaurs. Eleven synapomorphies support the monophyly of Therizinosauria in all of the most parsimonious trees and *Jianchangosaurus yixianensis* is assigned to Therizinosauria because it possesses five characters among the synapomorphies for this clade. The neural spines of the anterior caudal vertebrae are separated into anterior and posterior alae (character 117). The pubic boot has both anterior and posterior extensions, with the posterior extension being more pronounced (character 178), and the shelf on the pubic shaft proximal to the symphysis extends medially from the middle of the cylindrical pubic shaft (character 179). A rectangular buttress, which underlies the ventromedial surface of metacarpal II, is present on the ventrolateral side of the proximal end of metacarpal I (character 295). The cross-section of metatarsal shafts is rounded and not appressed (character 335). Three characters, suggested as synapomorphies of Therizinosauria by Zanno [2], are equivocal in this analysis. Two of these characters (prominent ventral depression on the cervical centra and prominent crests on the caudolateral margins of ventral cervical centra; characters 269 and 270) are absent in *Jianchangosaurus yixianensis,* although they are present in other therizinosaurs. The other character, a significantly expanded distal end of the humerus (character 293), is absent in *Jianchangosaurus yixianensis* as well as some therizinosaurids (*Segnosaurus galbinensis*, *Nothronychus graffami*, and *Nothronychus mckinleyi*).

The strict consensus tree shows that *Jianchangosaurus yixianensis* is more derived than *Falcarius utahensis* and a sister taxon to the Therizinosauroidea (the clade of *Beipiaosaurus inexpectus* and higher taxa) (Figure 12A), sharing five synapomorphies. Four of these characters are skull features. The premaxilla is edentulous (character 80). The symphyseal end of the dentary is downturned with a rostral gap (character 66). The dentary has subparallel dorsal and ventral borders in lateral view, but deepens posteriorly (character 70). The labial face of dentary has a lateral ridge, and the tooth row is inset from the main body of the dentary (character 69). The last character is present in the femur. The region bridging the femoral head and the greater trochanter region is constricted in dorsal view (character 321). In Zanno’s analysis, all characters except the edentulous premaxilla are shown as synapomorphies of Therizinosauroidea [2]. In *Jianchangosaurus yixianensis*, the dentary teeth, posterior to the tenth position, are inset from the lateral main surface of the dentary by a shelf (Figures 3A, B, 4A). The shelf is wide between the tenth to twenty-second dentary teeth. The anterior portion of the dentary, anterior to the shelf, is downturned; therefore there is a gap between the upper and lower jaws in the anterior portion. The premaxilla is edentulous, a condition only shared with *Erlikosaurus andrewsi*. These features may indicate that *Jianchangosaurus yixianensis* shows modifications in jaw arrangements for a more herbivorous diet than *Falcarius utahensis*.

*Jianchangosaurus yixianensis* is more basal to *Beipiaosaurus inexpectus*, the most basal member of Therizinosauroidea. The clade of Therizinosauroidea is supported by eight synapomorphies: nearly symmetrical dentary and maxillary teeth (excluding the rostral-most dentary teeth) in labial view (character 266); large serration denticles on the dentary and maxillary teeth (character 86); hooked denticles that point toward the tip of the caudal dentary and maxillary tooth crowns (character 87); lateral face of ischiadic blade with a longitudinal ridge (character 168); dorsal margin of ilium rises steeply, extending at least at a 30 degree angle from axis (character 307); hyperelongate pubic peduncle of ilium (character 311); greater trochanter and femoral head depressed in cranial view (character 339); fibular crest on tibia long, extending to midshaft (character 328). All of this demonstrates that *Jianchangosaurus yixianensis* is clearly different in characters of skull, dentition, pelvis and hind limbs from *Beipiaosaurus inexpectus* and more basal, despite being from the same formation as the latter.

## Discussion

*Jianchangosaurus yixianensis* is recovered from the same formation as the most basal known therizinosauroid, *Beipiaosaurus inexpectus*, but differs from it in some characters. Xu et al. [3] originally diagnosed *Beipiaosaurus inexpectus* mainly on limb element characters. In her study of Therizinosauria, Zanno [2] suggested that some of these characters are symplesiomorphies (e.g. relatively large skull, functionally tridactyl pes with proximally compressed first metatarsal, unexpanded preacetabular process of ilium, elongate manus, elongated tibia relative to femur, and proximally compressed metatarsus) and emended the diagnosis of *Beipiaosaurus inexpectus*. *Jianchangosaurus yixianensis* differs from *Beipiaosaurus inexpectus* in the following four features. Manual phalanx I-1 has a short lateral articular surface (Figure 8C, D). The ischium has a straight obturator process and less developed ischiac boot (Figure 9C). The femur shaft lacks a ridge on the anterior surface (Figure 10A). Our phylogenetic analysis also suggests that *Jianchangosaurus yixianensis* differs from *Beipiaosaurus inexpectus* in sharing some plesiomorphies with *Falcarius utahensis* (Figure 12A, B) (see Phylogenetic Analysis). Additional characters, which differentiate it from *Beipiaosaurus inexpectus* include a skull that is longer than the femur, taller tooth crowns, less curved manual phalanges, and a flexor tubercle on the manual unguals that is confluent with the phalangeal articular surface.

*Jianchangosaurus yixianensis* is unique among therizinosaurs because of five characters identified as autapomorphies based on phylogenetic analysis (Appendix 2 in File S1). The ventral surface of the cranial cervical vertebrae lacks hypapophyses (Figure 6A-C), whereas other therizinosaurs possess hypapophyses (character 102). Box-shaped centra in the cranial-most caudal vertebrae (character 117) are commonly present in maniraptorans (therizinosaurs, oviraptorosaurs, troodontids, and dromaeosaurids). *Jianchangosaurus yixianensis* is the only therizinosaur with oval shape caudal centra (Figure 7B, C). All manual unguals are weakly curved, with weak flexor tubercles ventral to the articular facet (character 151), a feature shared with *Therizinosaurus cheloniformis*. However, the unguals are much smaller and more curved than those of *Therizinosaurus* (Figure 8C, D). The participation of the jugal in margin of the antorbital fenestra (character 264) is common in coelurosaurs but rarely seen in other taxa such as *Garudimimus brevipes*, *Oviraptor mongoliensis* and *Oviraptor philoceratops* and is also absent in *Jianchangosaurus yixianensis* (Figure 3A, B). The shaft of the ulna is bowed in other basal therizinosaurs (*Falcarius utahensis* and *Alxasaurus elesitaiensis*) but not in *Jianchangosaurus yixianensis* (character 294). In therizinosaurids, three taxa (*Segnosaurus galbinensis*, *Therizinosaurus cheloniformis*, and *Nothronychus graffami*) have a straight ulna, but four others (*Falcarius utahensis*, *Alxasaurus elesitaiensis*, *Erliansaurus bellamanus*, and *Nothronychus mckinleyi*) have a bowed ulna. The straight ulna in *Jianchangosaurus yixianensis* may be reversal.

This study demonstrates that *Jianchangosaurus yixianensis* exhibits morphological characters related to feeding behavior, such as down turned anterior end of dentary with a gap and inset anterior teeth with a shelf, that are intermediate between *Falcarius utahensis* and *Beipiaosaurus inexpectus*, (Figure 3A, B). The edentulous premaxilla and a series of foramina along the buccal margin on the lateral surface may indicate that the anterior upper jaw of *Jianchangosaurus yixianensis* (and therizinosauroids) was covered with a rhamphotheca (Figure 2B), similar to ornithomimosaurs [27]–[29]. Derived features in the skull of *Jianchangosaurus yixianensis*, combined with primitive features in the postcrania (especially pelvis and hindlimbs), suggest that adaptations for herbivory in the cranium evolved before changes in the postcrania (e.g., large body cavity, wide pelvis, and robust hind limbs) [8], [11], similar to ornithomimosaurs [30] and pterosaurs [31].

The most striking feature of *Jianchangosaurus yixianensis* is the tooth arrangement in the middle and posterior portion of the dentary. Middle and posterior dentary teeth (more posterior to the seventh tooth) are offset medially from lateral border of the dentary by a shelf (Figures 3A, B, 4F). Middle and posterior dentary tooth crowns exhibit reversed tooth morphology, with a concave labial side (Figure 4D) and convex lingual side (Figure 4E), whereas the crowns of all maxillary teeth and six anterior dentary teeth have the normal condition, namely a convex labial side and concave lingual side (Figure 2B). The anterior portion of the upper jaw may have been covered by a rhamphotheca. The anterior portion of the lower jaw is down-turned and exhibits conventional tooth morphology (convex labial surface and concave lingual surface), which might have functioned to pluck food (e.g., plant material). The posterior portion, where the maxillary teeth have the opposite arrangement, so that the tips of the upper and lower teeth can abut each other, likely maximized the biting stress during occlusion to cut fibers of plant material, similar to ornithopods and ceratopsians. This line of evidence suggests that *Jianchangosaurus yixianensis* may have been adapted for herbivory in a different way than other therizinosaurs.

Co-occurrences of different therizinosaurs from the same formation are relatively common in the Upper Cretaceous deposits of Asia. The lower Upper Cretaceous Bayanshree Svita formation (Cenomanian-Santonian) of Mongolia has yielded three taxa: *Enigmosaurus mongoliensis*, *Segnosaurus galbinensis*, and *Erlikosaurus andrewsi* [18], [32], [33]. Recently, two additional taxa were described from the Campanian Iren Dabasu Formation of China: *Erliansaurus bellamanus* and *Neimongosaurus yangi* [22], [34]. Although *Alxasaurus elesitaiensis* and *Suzhousaurus megatheriodes* are not from the same rock unit, both are known from the Lower Cretaceous deposits of China (Aptian-Albian for the Bayin-Gobi Formation and Albian for Xinminpu Group, respectively) [4], [5]. The co-occurrence of *Jianchangosaurus yixianensis* and *Beipiaosaurus inexpectus* from the Yixian Formation suggests that co-occurrence of therizinosaurs is common in the Early Cretaceous as well, and that these therizinosaurs may have shared the same space by having a different strategy for feeding.

## Supporting Information

File S1 **Character states for Jianchangosaurus yixianensis, used in the phylogenetic analysis in this study and synapomorphies at each node in all of most parsimonious trees (** **Figure 12** **), obtained by TNT.**
(DOCX)
