# Supplementary material for: An Unusual Basal Therizinosaur Dinosaur with an Ornithischian Dental Arrangement from Northeastern China
Source: PLoS One. 2013 May 29;8(5):e63423. doi: 10.1371/journal.pone.0063423 (PMC3667168; doi:10.1371/journal.pone.0063423)
Supplement: File S1 — Character states for Jianchangosaurus yixianensis, used in the phylogenetic analysis in this study and synapomorphies at each node in all of most parsimonious trees ( Figure 12 ), obtained by TNT. (DOCX) [file pone.0063423.s001.docx]

Appendices

Appendix 1. Character states for *Jianchangosaurus yixianensis*, used in the phylogenetic analysis in this study.

??1?? ????? ????? ????1 0010? ?1000 ????? ?0?00 ????? ???00 00??? ????? ????0 10?11 0???? ??0?1 ?0010 1000? ?0010 01111 0001? 010?0 ???0? ?01?? ?0?00 1???? ??000 0?000 0100? ?0?00 20000 011?0 01?20 10120 200?0 00100 01000 000?? ????0 ????0 ???0? ???00 0?001 1?1?0 000?? 0???? 0010? 00000 0??0? 0001? ????? ??100 110?? 01?00 ??0?? 1010? ?010? 0?0?0 10001 ?0?0? ?00?0 ?00?0 01?0? 000?0 101?1 ??0?? ????0 ?0000 0?0?0 0?011

Appendix 2. Synapomorphies at each node in all of most parsimonious trees (Figure 12), obtained by TNT.

Therizinosauria: 13, 90, 113(2), 118, 137, 178, 179(0), 265, 275, 295, 304, 326, 335(0)

*Jianchangosaurus*+Therizinosauroidea: 66, 69, 70, 80, 215, 321

Therizinosauroidea: 86(0), 87, 168(2), 266, 307, 311(2), 328, 339

*Alxasaurus*+Therizinosauridae: 280, 300, 308, 320

*Jianchangosaurus*: 102(0), 117(0), 151(2), 246(0), 293(0)
